# Comparison of body mass index (BMI) with the CUN-BAE body adiposity estimator in the prediction of hypertension and type 2 diabetes

**DOI:** 10.1186/s12889-016-2728-3

**Published:** 2016-01-27

**Authors:** Vicente Martín, Verónica Dávila-Batista, Jesús Castilla, Pere Godoy, Miguel Delgado-Rodríguez, Nuria Soldevila, Antonio J. Molina, Tania Fernandez-Villa, Jenaro Astray, Ady Castro, Fernando González-Candelas, José María Mayoral, José María Quintana, Angela Domínguez, CIBERESP Cases and Controls in Pandemic Influenza Working Group, Spain

**Affiliations:** 1 Grupo de Investigación Interacciones Gen-Ambiente y Salud - Universidad de León (Gigas), León, Spain; 2 CIBER Epidemiología y Salud Pública, Madrid, Spain; 3 Instituto de Salud Pública de Navarra, Pamplona, Spain; 4 Departament de Salut, Generalitat de Catalunya, Barcelona, Spain; 5 División de Medicina Preventiva y Salud Pública, Universidad de Jaén, Jaén, Spain; 6 Subdirección de Vigilancia. Comunidad de Madrid, Madrid, Spain; 7 CIBER Enfermedades Respiratorias, Madrid, Spain; 8 Unidad Mixta Genómica y Salud CSISP (FISABIO)-Universitat de València, Valencia, Spain; 9 Servicio de Vigilancia de Andalucía, Sevilla, Spain; 10 Fundación Vasca de Innovación e Investigación Sanitarias, Sondika, Spain; 11 Departament de Salut Pública, Universitat de Barcelona, Barcelona, Spain; 12 Facultad de Ciencias de la Salud. Campus de Vegazana. Universidad de León, 24071 León, Spain

**Keywords:** Obesity, Body mass index, Body fat, CUN-BAE, Population attributable fraction, Hypertension, Diabetes mellitus

## Abstract

**Background:**

Obesity is a world-wide epidemic whose prevalence is underestimated by BMI measurements, but CUN-BAE (Clínica Universidad de Navarra - Body Adiposity Estimator) estimates the percentage of body fat (BF) while incorporating information on sex and age, thus giving a better match. Our aim is to compare the BMI and CUN-BAE in determining the population attributable fraction (AFp) for obesity as a cause of chronic diseases.

**Methods:**

We calculated the Pearson correlation coefficient between BMI and CUN-BAE, the Kappa index and the internal validity of the BMI. The risks of arterial hypertension (AHT) and diabetes mellitus (DM) and the AFp for obesity were assessed using both the BMI and CUN-BAE.

**Results:**

3888 white subjects were investigated. The overall correlation between BMI and CUN-BAE was R^2^ = 0.48, which improved when sex and age were taken into account (R^2^ > 0.90). The Kappa coefficient for diagnosis of obesity was low (28.7 %). The AFp was 50 % higher for DM and double for AHT when CUN-BAE was used.

**Conclusions:**

The overall correlation between BMI and CUN-BAE was not good. The AFp of obesity for AHT and DM may be underestimated if assessed using the BMI, as may the prevalence of obesity when estimated from the percentage of BF.

## Background

Obesity is seen as an emerging epidemic around the world because it represents a growing threat to the health of the population. It is a complex disease consisting of an excess or abnormal distribution or both of adipose tissue, giving rise to metabolic and endocrine alterations and changes in the immune system, resulting in increased morbidity and mortality and a lower life expectation [1, 2]. Moreover, excess body fat (BF) is known to be associated with cardiovascular diseases and diabetes [3].

The body mass index (BMI) is the most frequently used measurement for diagnosing obesity, because of its simplicity and reliability. However, the BMI underestimates the prevalence of obesity by 50 %, in comparison with direct measurement techniques of adipose; its relationship with adiposity is influenced by age, sex and race [1, 4–7].

In this regard, an alternative for whites is the CUN-BAE (Clínica Universidad de Navarra - Body Adiposity Estimator), which gives a closer correlation between adiposity and cardiovascular factors than BMI, improving our understanding of the impact of obesity levels on these chronic diseases [8].

Our aim is to compare the BMI and CUN-BAE and evaluation the population attributable fraction (AFp) for obesity as a cause of hypertension and type 2 diabetes.

## Methods

### Population studied

The present study incorporated all the white patients taking part in the cross-sectional project concerning the Risk Factors of Infuenza A(H1N1) in the 2009–10 and 2010–11 seasons aged over eighteen with a BMI ≥ 18.5 kg/m^2^, with the exception of pregnant women. The project involved twenty-nine hospitals in seven Spanish autonomous regions and nine research groups in CIBERESP, the Spanish Consortium for Biomedical Research in Epidemiology and Public Health [9].

### Anthropometrical measurements

The body mass index (BMI) was calculated in the standard way as kg/m^2^. Patients were classified by BMI according to the criteria of the World Health Organization (WHO) and the Spanish Society for the Study of Obesity, with obesity being taken to be a BMI of 30 kg/m^2^ or more for both sexes [10, 11].

The CUN-BAE figure was then calculated, using the following equation [8]:

$$ \begin{array}{l}\%\ BF = \mathit{\hbox{-}} 44.988 + \left( 0.503 \times age\right) + \left( 10.689 \times sex\right) + \left( 3.172 \times BMI\right)\ \mathit{\hbox{-}}\ \left( 0.02 6 \times BM{I}^2\right) + \\ {}\ \left( 0.181 \times BMI \times sex\right)\ \mathit{\hbox{-}}\ \left( 0.02 \times BMI \times age\right)\ \mathit{\hbox{-}}\ \left( 0.005 \times BM{I}^2 \times sex\right) + \left( 0.00021 \times BM{I}^2 \times age\right)\end{array} $$

where age was in years, and sex was coded as 0 for men and 1 for women. Obesity was taken to be a percentage of BF ≥ 25 % in males and ≥ 35 % in women, increments of 5 % being used to divide categories [8, 12, 13].

Subjects were defined as hypertensive (AHT) or as having type 2 diabetes mellitus (DM) if they had previously been diagnosed for either.

### Statistical analyses

Agreement between BMI and CUN-BAE was assessed by means of the Pearson correlation coefficient. The Kappa coefficient and its index of coincidence at 95 % were calculated so as to classify patients as obese or not using both methods of determining obesity. All the analyses involved grouping by sex and into the two age bands of under 50 and 50 plus.

Association of type 2 diabetes mellitus (DM) or arterial hypertension (AHT) to BF was assessed using the two methods for calculating body fat. The comparative standard adopted was the normal weight category [2, 8], and the level of risk (crude odds ratio, cOR) was calculated for each of the distribution categories. By means of a logistic regression model adjusted odds ratio (aOR) figures were reckoned for the risk of AHT and DM by including in the model details of education, marital status and tobacco and alcohol use. Age was factored into the BMI analyses, but not into CUN-BAE, which already includes it. All these analyses were grouped by sex.

Calculation of the population attributable fraction (AFp) for AHT and DM in the BMI and CUN-BAE categories was on the basis of the following formula expressed as a percentage [14]:

$$ 1-{\displaystyle {\sum}_l^k\left(\mathrm{p}\mathrm{d}/aOR\right)} $$

where pd is the proportion of those suffering from the ailments at the level of exposure, and aOR is the adjusted odds ratio.

Data analysis was performed with the Stata/SE 13 software package.

### Data confidentiality and ethical considerations

All information collected was treated as confidential under the observational studies law. The study was approved by the Ethics Committee of the hospitals involved: Clinical Research Ethics Committee, Hospital Costa del Sol; Autonomous Clinical Trials Committee of Andalusia; Clinical Research Ethics Committee, Complejo Asistencial Universitario de León; Clinical Research Ethics Committee, Municipal Institute of Healthcare (CEIC-IMAS); Clinical Research Ethics Committee, Corporación Sanitaria ParcTaulí of Sabadell; Research Committee, Sant Joan de Déu University Hospital; Clinical Research Ethics Committee, Basque Country; Clinical Research Ethics Committee, Doctor Peset Univeristy Hospital, Valencia; and, Clinical Research Ethics Committee, Clinical Research Ethics Committee, General Directorate of Public Health, Valencia. Written informed consent was obtained from all patients.

## Results

A total of 3888 patients were studied: 2033 men with an average age of 50.7 years, and 1855 women with an average age of 49.6 years. The average BMI was 26.9 kg/m^2^ for the men and 26.3 kg/m^2^ for the women. The average CUN-BAE was 27.1 % of BF for the men and 37.6 % for the women.

**Fig. 1:**
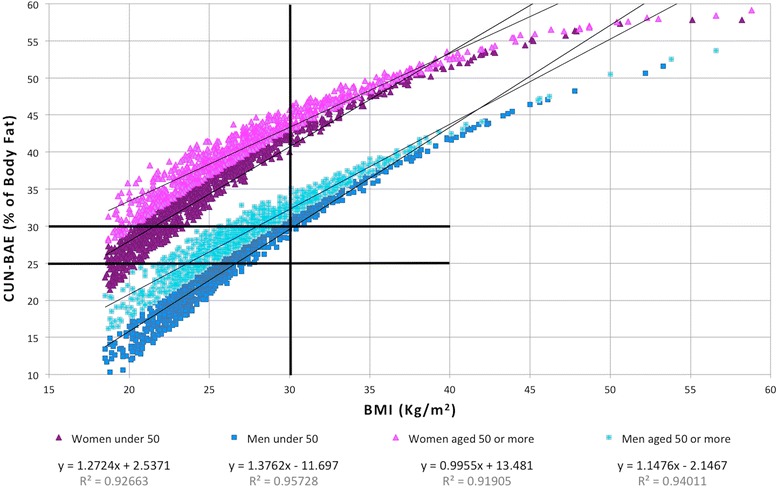
Distribution for CUN-BAE and BMI. Straight-Line Equation and Correlation by Sex and Age

Figure 1 shows the distribution of BMI and CUN-BAE. From the four groupings depending on sex and age. The correlation between BMI and CUN-BAE was low (R^2^ = 0.48), but increased considerably when sex was taken into account (R^2^ above 0.88 in both sexes). This improvement was even greater when age (under 50 or 50 plus) was also considered, when R^2^ was greater than 0.92 (Table 1).

**Table 1:** Correlation and degree of agreement between CUN-BAE and BMI and prevalence of obesity according to sex and age groups

	N	R2	Obesity^a^				Level of Agreement	Kappa	Kappa(%) CI 95 %	
			BMI		CUN-BAE					
			n	%	n	%				
Men										
Total	2033	0.877	435	21.4	1254	61.7	59.7	28.9	26.3	31.6
Under 50	971	0.957	183	18.8	412	42.4	76.4	47.9	42.9	53.0
50 or over	1062	0.940	252	23.7	842	79.3	44.4	15.0	12.6	17.5
Women										
Total	1855	0.915	395	21.3	1150	62.0	59.3	28.5	25.7	31.2
Under 50	921	0.926	134	14.5	367	39.8	74.7	40.9	35.6	46.2
50 or over	934	0.919	261	27.9	783	83.8	44.1	13.9	11.4	16.4

^a^Obesity: BMI ≥ 30 kg/m2, CUN-BAE ≥25 % body fat in men and 25 % in women

Abbreviations: *R2* square of the sample correlation coefficient, *BMI* body mass index, *CUN-BAE* Clínica Universidad de Navarra - Body Adiposity Estimator

The degree of agreement measured by the Kappa coefficient for diagnosis of obesity was low (28.7 %), similar for both sexes and somewhat better for those under 50 than the others. This low level of agreement with BMI explains the different prevalence of obesity noted in accordance with the criterion used. In all cases the prevalence of obesity as based on the estimation of body fat CUN-BAE is three times higher than the BMI would suggest.

**Fig. 2:**
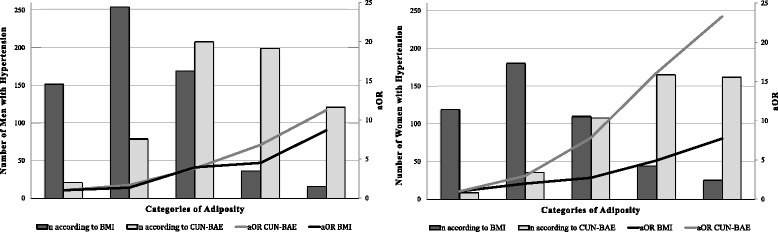
Distribution of Number of Cases of hypertension and aOR, by sex. Legends: Categories of Adiposity: With BMI (C1: 18,5–24.9, C2: 25–29.9, C3: 30–34,9, C4: 35–39.9, C5: ≥ 40 Kg/m2) with CUN-BAE men (C1: ≤ 19.9, C2: 20–24.9, C3: 25–29.9, C4: 30–34.9, C5: ≥ 35 %BF) and women (C1: ≤ 29.9, C2: 30–34.9, C3: 35–39.9, C4: 40–44.9, C5: ≥ 45 %BF)

Table 2 and Fig. 2 show that as the figures for both BMI and CUN-BAE increased, so did the prevalence of AHT and the aOR values. However, this gradient was more evident with CUN-BAE than with BMI, basically owing to a lesser prevalence of AHT in the normal weight group based on the criterion of estimated body fat (8.1 % and 3.0 % in men and women respectively) than when based on BMI (20.3 % and 13.3 % in men and women respectively). The AFp of AHT assigned to the two methods of assessing adiposity was found to be double for both men and women for CUN-BAE in comparison with BMI (37.0 % and 45.4 % with BMI; 74.0 % and 89.1 % with CUN-BAE for men and women, respectively). In men this difference is due to the differing distributions of cases with the two methods of evaluating adiposity, and a mixture of this and differences in risks in the case of women.

**Table 2:** Distribution of prevalence and risk of hypertension by sex according to BMI and CUN-BAE

	N	n	Prev	cOR	CI 95 %	aOR^a^	CI 95 %	AFp(%)
Men								
BMI (kg/m2)								
18.5–24.9	749	152	20.29	1		1		37.00
25–29.9	849	254	29.92	1.68	1.33–2.11	1.33	1.02–1.74	
30–34.9	328	169	51.52	4.17	3.15–5.53	3.93	2.84–5.44	
35–39.9	74	37	50.00	3.93	2.40–6.40	4.50	2.56–7.89	
≥40	33	16	48.48	3.70	1.83–7.49	8.66	3.80–19.39	
CUN-BAE (%BF)								
≤19.9	258	21	8.14	1		1		74.03
20–24.9	521	79	15.16	2.02	1.22–3.35	1.67	1.00–2.80	
25–29.9	631	208	32.96	5.55	3.45–8.93	3.80	2.32–6.21	
30–34.9	415	199	47.95	10.40	6.40–16.90	6.80	4.11–11.25	
≥35	208	121	58.17	15.70	9.29–26.52	11.24	6.54–19.31	
Women								
BMI (kg/m2)								
18.5–24.9	895	119	13.30	1		1		45.32
25–29.9	565	180	31.86	3.05	2.35–3.96	1.99	1.46–2.71	
30–34,9	259	110	42.47	4.81	3.52–6.58	2.73	1.87–3.98	
35–39.9	86	45	52.33	7.15	4.50–11.39	4.93	2.83–8.58	
≥40	50	26	52.00	7.06	3.93–12.71	7.71	3.80–15.64	
CUN-BAE (%BF)								
≤29.9	303	9	2.97	1		1		89.14
30–34.9	402	36	8.96	3.21	1.52–6.78	2.97	1.34–6.57	
35–39.9	464	108	23.28	9.91	4.93–19.90	7.82	3.71–16.49	
40–44.9	389	165	42.42	24.06	12.03–48.12	16.10	7.63–33.99	
≥45	297	162	54.55	39.20	19.44–79.06	23.30	10.93–49.69	

^a^aOR: BMI (age, educational level, marital status, tobacco and alcohol use); CUN-BAE (educational level, marital status, tobacco and alcohol use)

Abbreviations: *CI* confidence interval, *aOR* adjusted odds ratio, *AFp* population attributable fraction, *BMI* body mass index, *CUN-BAE* Clínica Universidad de Navarra - Body Adiposity Estimator, *BF* body fat

**Fig. 3:**
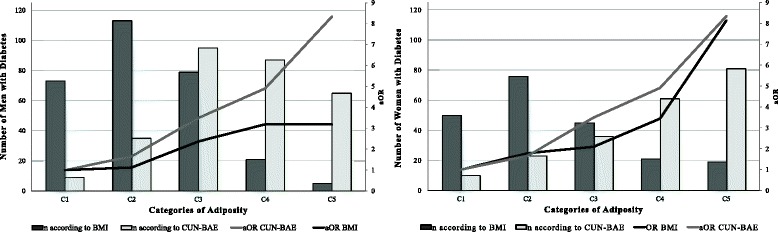
Distribution of the Number of Cases of Diabetes and aOR, by sex. Legends: Categories of Adiposity: With BMI (C1: 18,5–24.9, C2: 25–29.9, C3: 30–34,9, C4: 35–39.9, C5: ≥ 40 Kg/m2) with CUN-BAE men (C1: ≤ 19.9, C2: 20–24.9, C3: 25–29.9, C4: 30–34.9, C5: ≥ 35 %BF) and women (C1: ≤ 29.9, C2: 30–34.9, C3: 35–39.9, C4: 40–44.9, C5: ≥ 45 %BF)

Table 3 and Fig. 3 show how prevalence and aOR figures for DM increase according to the category of obesity in both sexes for CUN-BAE and in women for BMI. This gradient is more obvious with CUN-BAE than with BMI for men and similar for the two among women, although they yield different distributions. Regarding the AFp of DM the CUN-BAE almost tripled the attributable percentage in comparison with BMI in men (71.54 % as opposed to 26.19 %), while for women the figure was 50 % higher (65.19 % as opposed to 40.38 %).

**Table 3:** Distribution of prevalence and risk of diabetes II by sex according to BMI and CUN-BAE, 2009–2011

	N	n	Prev	cOR	CI 95 %	aOR^a^	CI 95 %	AFp(%)
MEN								
IMC								
18.5–24.9	749	73	9.75	1		1		26.19
25–29.9	849	113	13.31	1.42	1.04–1.94	1.13	0.80–1.58	
30–34,9	328	79	24.09	2.94	2.07– 4.17	2.37	1.62–3.48	
35–39.9	74	21	28.38	2.97	1.80–4.92	3.19	1.83–5.55	
≥40	33	5	15.15					
CUN-BAE								
≤19.9	258	9	3.49	1		1		71.54
20–24.9	521	35	6.72	1.99	0.94–4.21	1.66	0.78–3.55	
25–29.9	631	95	15.06	4.90	2.44–9.87	3.49	1.70–7.16	
30–34.9	415	87	20.96	7.34	3.62–14.86	4.91	2.37–10.14	
≥35	208	65	31.25	12.58	6.08–26.01	8.34	3.96–17.56	
WOMEN								
IMC								
18.5–24.9	895	50	5.59	1		1		40.38
25–29.9	565	76	13.45	2.63	1.81–3.82	1.78	1.20–2.64	
30–34,9	259	45	17.37	3.55	2.31–5.46	2.09	1.33–3.31	
35–39.9	86	21	24.42	5.46	3.09–9.64	3.44	1.86–6.35	
≥40	50	19	38,0	10.36	5.47–19.61	8.13	4.08–16.18	
CUN-BAE								
≤29.9	303	10	3.3	1		1		65.19
30–34.9	402	23	5.72	1.78	0.83–3.79	1.64	0.76–3.54	
35–39.9	464	36	7.76	2.46	1.20–5.04	1.86	0.89–3.87	
40–44.9	389	61	15.68	5.45	2.74–10.83	3.47	1.69–7.13	
≥45	297	81	27.27	10.99	5.57–21.69	6.49	3.16–13.3	

^a^aOR: BMI (age, educational level, marital status, tobacco and alcohol use); CUN-BAE (educational level, marital status, tobacco and alcohol use)

Abbreviations: *CI* confidence interval, *aOR* adjusted odds ratio, *AFp* population attributable fraction, *BMI* body mass index, *CUN-BAE* Clínica Universidad de Navarra - Body Adiposity Estimator, *BF* body fat

## Discussion

The correlation found between BMI and CUN-BAE in overall analyses was not good (R^2^ = 0.48). This coincides with the findings of Romero-Corral et al. and Sardinhha et al., who also noted a low overall agreement (R^2^ of 0.40 to 0.47) between BMI and the percentage of BF assessed by bio-electric impedance [7, 15]. This poor correlation is explained by the fact that adiposity is dependent upon sex and age. It is well known that with the same BMI women and more elderly subjects have a greater percentage of BF [15–18]. This same fact also explains the improvement in correlation when sex, age, or both are factored into the figures. This was also observed by Gallagher et al., who found an overall correlation R^2^ = 0.26, but when sex and age were taken into account the correlation was much better, with values for R^2^ of up to 0.67 [18]. On this point, it should be noted that there was a good coincidence between the correlation figures obtained in studies comparing BMI with directly measured body fat and body fat estimated with CUN-BAE [8, 18–20].

Regarding the classification of obesity, this study opted for BMI ≥ 30 kg/m^2^, regardless of sex or age, since this is the criterion recommended by the WHO, the most extensively used world-wide and by scientific associations in Spain [2, 11]. The cut-off point for CUN-BAE was based on the criteria indicated by the authors who described the formula for calculating it, and was thus coincident with recommendations in other studies [12, 21]. On the basis of these norms, there is a low level of agreement in classification of obesity between BMI and the percentage of BF estimated by CUN-BAE, with a Kappa coefficient of 28.7 %. In other publications the Kappa between BMI and percentage of BF was similarly low in women (between 15 % and 30 %), while in men greater variations were noted (between 8 % and 70 %).

In addition to BMI as compared to the percentage of BF, prevalence of obesity estimated with CUN-BAE (61.8 %) was much higher than with BMI (21.35 %). This coincided with other publications in which the prevalences of obesity estimated through the percentage of BF were almost double those yielded by BMI [4, 22] or even up to six times higher [23].

Diabetes and AHT are common ailments clearly related to obesity as a risk factor, which is why we studied their association with the two ways of assessing body fat, to find that estimates of BF according to CUN-BAE were more clearly related to AHT and DM than results from BMI, just as was noted by Dervaux et al. in the assessment of body fat percentages [24]. The main reason for this clearer association lies in the lower prevalence of AHT and DM in the normal weight grouping as assigned on the criterion of estimated body fat than as assigned by BMI. Furthermore, the greater number of instances of AHT and DM are to be found in lower-weight categories according to BMI, while with CUN-BAE they are present in a smaller number of individuals. Other studies have also shown a better correlation of CUN-BAE with other biological markers of cardiovascular and metabolic diseases [8, 25].

The final result, the disparity in aOR and essentially in the distribution of patients according to BMI or CUN-BAE, comes down to the great differences observed in the attributable portion of the population for AHT or DM on the basis of quantity of body fat. Indeed, the fact that the majority of patients had high percentage of BF, while with BMI they were assigned to lower categories, goes a long way towards explaining the total number of cases attributed to higher than normal weight. In almost all instances the classification of patients according to CUN-BAE almost doubled the AFp relative to classification in accordance with BMI. It may also be of some relevance that the reference group with BMI (normal weight) is a very broad grouping in which risk may be expected not to be homogeneous, in the sense that individuals in the upper part of the range might present a risk more like that of the over-weight than that of the lower part of the normal weight spectrum. All of this may cast a doubt upon estimates made of portions or fractions of the population and cases of AHT and DM attributable to obesity as a function of BMI [26, 27], so that the real impact of obesity in these pathologies may be much greater than assumed.

CUN-BAE has been proposed as a substitute for the BMI. Few studies have assessed its usefulness for classifying obesity or determining obesity-related cardiovascular risks. Nevertheless such studies as have been carried out report the same phenomenon as we do here, showing that CUN-BAE classes a greater number of subjects as obese and therefore greatly reduces the number of individuals in the reference category [28–30].

One possible limitation is that the highest adiposity category in men was established to avoid a sample size problem. Furhtermore, in our findings the sample may not have been representative of the population as a whole: the subjects were patients admitted to hospital or making use of health services for various reasons, so the prevalence of obesity and of AHT and DM was higher than in the population in general [31, 32]. However, the aOR observed in relation to BMI for AHT and DM was very similar to that reported in another study [26].

## Conclusions

Although the overall correlation between BMI and the BF estimator was not good, it improved when sex and age were taken into account.

There is a low level of agreement in accordance with the criterion used. The prevalence of obesity as based on estimation of body fat is the three times higher than the BMI would suggest, which could lead to an underestimation of the prevalence of obesity.

CUN-BAE showed links with hypertension and diabetes mellitus, and presented a better gradient than BMI did. The AFp for AHT was double when assessed with CUN-BAE as compared to BMI, while for DM it was more than 50 % higher. This brings into question the reliability of calculations undertaken to assess the impact of obesity on thesepathologies.

## Abbreviations

AFp: population attributable fraction
AHT: arterial hypertension
BF: body fat
BMI: body mass index
CUN-BAE: Clínica Universidad de Navarra - Body adiposity estimator
DM: diabetes mellitus
WHO: World Health Organization
